# Impact evaluation of a community engagement intervention in improving childhood immunization coverage: a cluster randomized controlled trial in Assam, India

**DOI:** 10.1186/s12889-018-5458-x

**Published:** 2018-04-23

**Authors:** Santanu Pramanik, Arpita Ghosh, Rituu B. Nanda, Marlou de Rouw, Philip Forth, Sandra Albert

**Affiliations:** 1 Public Health Foundation of India, Plot 47, Sector 44, Institutional Area, Gurgaon, Haryana 122002 India; 2 The Constellation, Sentier des Cinq Bonniers 25, 1390 Grez Doiceau, Belgium; 3 Indian Institute of Public Health, Shillong (IIPH-S), Lawmali, Pasteur Hill, Shillong, Meghalaya 793001 India

**Keywords:** Community participation, SALT, Ownership, Demand-side intervention, North-East India, Universal immunization program, Full immunization, DPT3, Randomized evaluation, Study protocol

## Abstract

**Background:**

To improve immunization coverage, most interventions that are part of the national immunization program in India address supply-side challenges. But, there is growing evidence that addressing demand-side factors can potentially contribute to improvement in childhood vaccination coverage in low- and middle-income countries. Participatory engagement of communities can address demand-side barriers while also mobilizing the community to advocate for better service delivery. The objective of this study is to evaluate the impact of a novel community engagement approach in improving immunization coverage. In our proposed intervention, we go a step beyond merely engaging the community and strive towards increasing ‘ownership’ by the communities.

**Methods/Design:**

We adopt a cluster randomized design with two groups to evaluate the intervention in Assam, a state in the northeast region of India. To recruit villages and participants at baseline, we used a two-stage stratified random sampling method. We stratified villages; our unit of randomization, based on census data and randomly selected villages from each of the four strata. At the second-stage, we selected random sub-sample of eligible households (having children in the age group of 6–23 months) from each selected village. The study uses a repeated cross sectional design where we track the same sampled villages but draw independent random samples of households at baseline and endline. Total number of villages required for the study is 180 with 15 eligible HHs from each village. Post-baseline survey, we adopt a stratified randomization strategy to achieve better balance in intervention and control groups, leveraging information from the extensive baseline survey.

**Discussion:**

The proposed intervention can help identify barriers to vaccination at the local level and potentially lead to more sustainable solutions over the long term. Our sampling design, sample size calculation, and randomization strategy address internal validity of our evaluation design. We believe that it would allow us to causally relate any observed changes in immunization coverage to the intervention.

**Trial registration:**

The trial has been registered on 7th February, 2017 under the Clinical Trials Registry- India (CTRI), hosted at the ICMR’s National Institute of Medical Statistics, having registration number CTRI/2017/02/007792. This is the original study protocol.

## Background

Immunization is a globally accepted public health intervention that helps avert vaccine-preventable diseases. Incomplete- and non-immunization increases the risk of illness and death among children. The Global Vaccine Action Plan (GVAP 2011–2020) sets the goal of 90% coverage at the national level and 80% in every district or equivalent administrative unit with all vaccines in national programs by 2020 [1]. India is one of the 194 Member States of the World Health Assembly which endorsed the GVAP framework in 2012. Despite a long standing national program for immunization in India since 1985, only 65.2% of 12–23 month old children are fully immunized (RSOC, 2013–14) [2]. Full immunization is defined as children receiving one dose of Bacillus Calmette–Guérin (BCG) vaccine to prevent tuberculosis, three doses of oral polio vaccine (OPV3), three doses of diphtheria-pertussis-tetanus vaccine (DPT3) or the more recently introduced Pentavalent (DPT-Hepatitis B-Haemophilus Influenzae type B) vaccine, and one dose of measles vaccine. Completion of schedule of vaccines that require multiple doses (e.g., OPV and DPT or Pentavalent) remains a major challenge towards achieving higher full immunization coverage (FIC). Both demand- and supply-side bottlenecks contribute to sub-optimal vaccination coverage rates in India [3–8].

In the context of universal immunization program (UIP) in India, most of the existing interventions are geared towards addressing supply-side challenges such as ensuring better immunization services and more focused implementation by deploying more health workers across health facilities, introducing alternate vaccine delivery system, including new vaccines in the immunization schedule, organizing sessions in hard-to-reach areas, and initiating supplementary immunization activities for children who are missed out in the routine immunization program [5, 9, 10]. But there is a growing body of literature showing that demand-side interventions lead to significant improvement in childhood vaccination coverage in low- and middle-income countries [11, 12]. Community engagement approaches can address demand-side barriers while also mobilizing the community to advocate for better service delivery [13, 14]. With the growing realization that community-level factors influence vaccination uptake, more recent strategies to increase vaccination coverage have attempted to focus on community-based interventions [15–18].

Existing community engagement programs, however, mostly focus on communication activities that do not actively involve communities in the planning, monitoring and surveillance activities [15]. Participatory engagement of communities can help identify barriers to vaccination at the local level and thus might lead to sustainable solutions in a manner which a top-down approach cannot achieve. Our proposed intervention goes a step beyond merely engaging with communities and strives towards increasing a sense of community ‘ownership’.

In various steps of the proposed intervention, referred to by the acronym SALT (Stimulate, Appreciate, Learn, Transfer), the communities lead the way — they identify the challenges, they take actions based on where they are and what they want to achieve, learn from their actions and share their experience with other communities. Trained facilitators stimulate the communities to leverage their own strengths to address their concerns, and accompany them through a systematic process of learning from action.

This community-based approach has been shown to be effective in generating behaviour change in other contexts. The WHO-UNICEF evaluation of AIDS Competence Process (ACP) in Papua New Guinea (2009) concludes that ACP is an effective approach in combating HIV/AIDS through local empowerment [19]. Economic evaluation of ACP in Thailand (2011) shows that it is likely to be cost-effective when comparing incremental cost-effectiveness ratio of the ACP with other HIV prevention programs [20]. In the context of malaria, a retrospective study of SALT versus non-SALT districts in Togo shows significant increase in impregnated bednet use and decrease in malaria prevalence among children under five [21]. A recent study (unpublished) in Democratic Republic of the Congo found that SALT intervention can reduce resistance to vaccination and increase vaccination rates for oral polio vaccine [22]. However, the evaluation design and method used in the study did not measure the attributable impact of SALT.

In the last 5–6 years, according to the different health and demographic surveys, there has been no significant improvement in the FIC in India [2, 4, 23]. It has been suggested that approaches in which communities play a prominent role may be effective for improving immunisation coverage [15]. We aim to assess the impact of this novel community engagement approach (SALT) in increasing immunization coverage. The objective of this impact evaluation study is to generate high quality evidence that will allow us to causally attribute the changes in immunisation coverage, if any, to the SALT intervention.

## Methods/Design

### Study setting

**Fig. 1:**
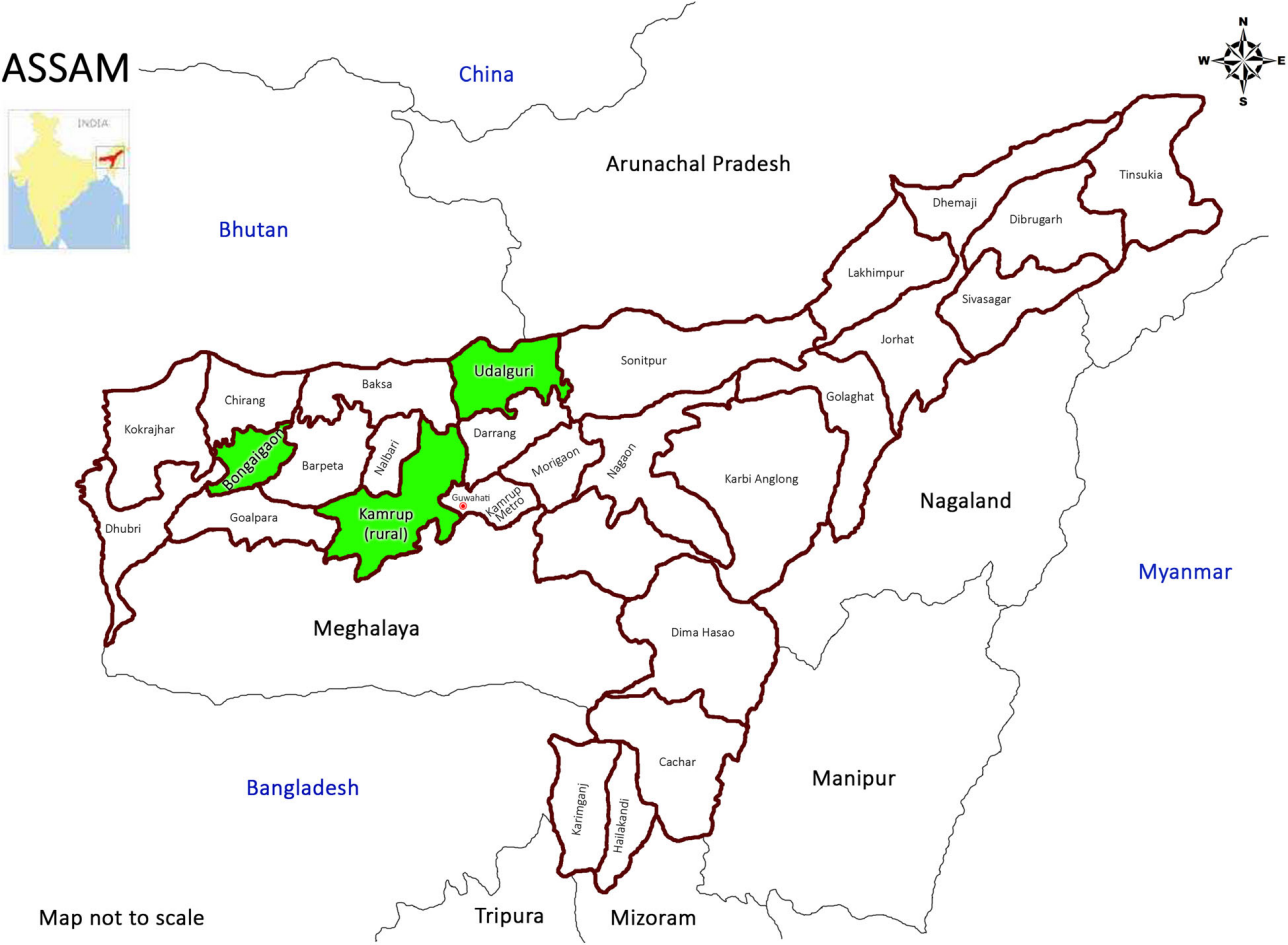
Location of Assam on India map (inset) and three selected districts- Bongaigaon, Kamrup (rural) and Udalguri- on Assam map. Source: https://www.mapsofindia.com/maps/assam/

**Fig. 2:**
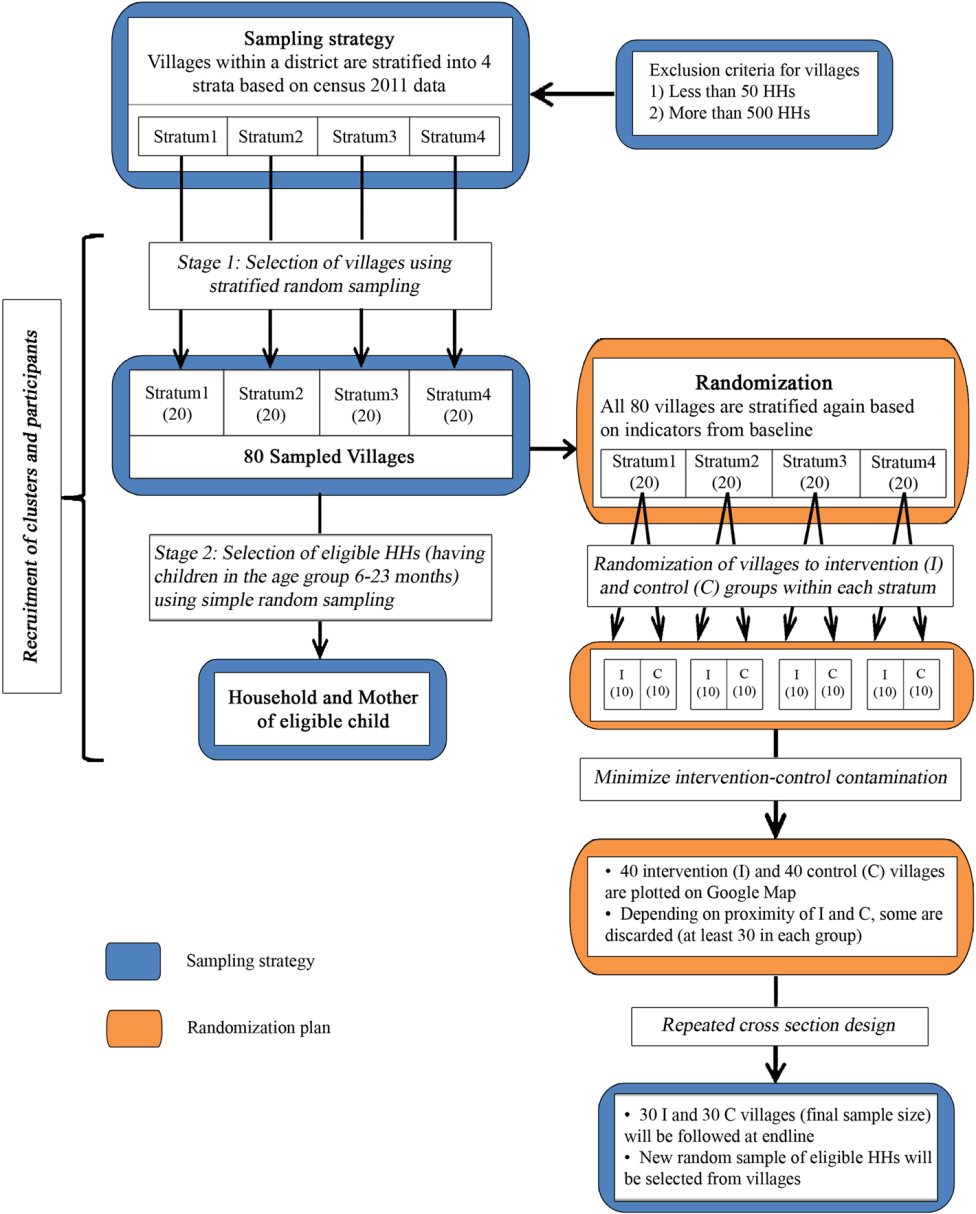
Flow chart describing sampling strategy and randomization plan for the cluster randomized controlled trial within a district: Exact same flow chart is applicable for all three districts

The study site is in Assam, a north-eastern state of India. Historically, Assam is known to have poor public health indicators and weak health infrastructure [24]. Full immunization coverage in Assam (55.3%) is lower than the national level (65.2%). Dropout rates for vaccines that require multiple doses are higher in Assam than the national figures [2]. According to the latest SRS bulletin (2015) [25], Assam has the second highest infant mortality rate in the country, 47 deaths in infants less than a year old per 1000 live births, which exceeds the national average of 37. We considered 3 districts from Assam- Bongaigaon, Kamrup rural, and Udalguri. Figure 1 shows the location of Assam on India map and the three selected districts on a map of Assam.

Stratified purposeful sampling was used so that the selected districts represent varied socio-demographic characteristics of Assam. Table 1 illustrates the variation across the three districts in terms of selected indicators. We considered only rural areas of selected districts for this impact evaluation study.

**Table 1:** Socio-demographic characteristics (census 2011) of three selected districts of Assam along with DPT3 coverage rate (DLHS-3, 2007–08)

District	Population	Urban (%)	Tribal (%)	Female literacy rate	Muslim (%)	DPT3 coverage
Bongaigaon	7,38,804	14.9	2.5	64.4	50.2	56.2
Kamrup rural	15,17,542	9.4	12.0	69.5	39.7	67.2
Udalguri	8,31,668	4.5	32.1	58.0	12.7	62.0

### Study design

We adopted a cluster randomized controlled trial (cRCT) design with two groups to evaluate the intervention. The intervention group receives SALT intervention for about a year (March 2017–March 2018) along with the routine immunization (RI) services. The control group received RI services alone. Cluster randomized design is appropriate here as opposed to individual or household level randomization as the intervention is intended for implementation at the community (village) level, the village being our unit of randomization.

The study uses a repeated cross sectional design where we track the same sampled villages but draw independent random samples of households (HHs) at baseline and endline. Post intervention, for the endline survey we do not plan to follow up the same HHs unlike most other RCTs, as estimating immunization coverage rates requires targeting a particular age-group of children. The children sampled at baseline would have mostly grown beyond the desirable age limit by the time the endline survey is due. The HHs in the baseline may no longer satisfy the eligibility criteria (having children in that particular age group) at the time of the endline survey – although there may be some overlap.

### Sampling strategy: Recruitment of clusters and participants

Within each district, we considered a two-stage cluster sampling design. In the *first stage*, we selected villages using a stratified sampling technique. We stratified villages, within a district into four strata and then randomly selected villages from each stratum. For the selection of villages, we used census 2011 village-level data as our sampling frame. The following villages were excluded from the census sampling frame: 1) villages having less than 50 HHs as getting enough number of eligible HHs–having children in the age group 6 to 23 months- would be a challenge and 2) villages having more than 500 HHs as there would be logistical challenges to get the community together for implementation within the timeframe and resources of the study. To stratify the villages, we used the following village-level indicators: population size, percentage of Scheduled Caste (SC), percentage of Scheduled Tribe (ST), and female literacy rate. SCs and STs are various officially designated groups, recognized in the constitution of India, of historically disadvantaged people [26]. Based on these indicators we developed a composite index that was then used to construct the four strata based on the quartiles of the index. Twenty villages were selected from each of the four strata using simple random sampling method. The choice of stratified sampling design ensures a representative sample of villages, having varied socio-demographic characteristics, within each district. The requirement of 20 villages per stratum stems from the sample size calculation, as discussed in the section on Sample Size. This led to 80 villages (20 × 4) in each district, resulting in total 240 villages across 3 districts of Assam for the baseline survey.

In the *second stage*, from all sampled villages, after houselisting a random sample of 15 eligible HHs was selected for the baseline survey. In a selected household, all mothers having children in the age group 6–23 months were eligible to participate in the survey. From mothers, we collected information pertaining to her youngest 6–23 month old child. The flowchart in Fig. 2 provides a visual display of the sampling strategy (in blue). In the endline survey, while the sampled villages will remain the same, a new random sample of 15 eligible HHs will be drawn from each village. The baseline survey was done during June–August, 2016 and the endline survey is planned to be conducted after the conclusion of the intervention, during June–August 2018.

### The intervention

This community engagement intervention originated at the Constellation [27], a NGO registered in Belgium. We refer to the intervention by the acronym SALT: stimulate, appreciate, learn, and transfer. The intervention starts with home visits by trained facilitators to villages. Facilitators engage with people, listen to the hopes and concerns of people, appreciate their strengths, and eventually bring the community together to discuss the common values they share. SALT home visits are a crucial starting point as it helps to build trust between the facilitators and the community and to identify strengths available in the community. The next step is collective dream building which starts from individuals and small groups and then involves the wider community. In our context, this is the dream of healthy children in the community and immunization is a component that can contribute to healthy children.

Once the community embraces its dream, a self-assessment exercise starts under the guidance of the facilitator, in order to understand where the community stands today with respect to practices linked to their shared dream. It is important to note that the practices must ideally come from the community. The facilitators stimulate the conversation so that immunization related practices emerge during self-assessment. The self-assessment framework requires the community to assess itself on the set of practices defined by the community from level 1 to level 5 where level 1 indicates a low level of competence and level 5 indicates a high level of competence. The central idea behind the self-assessment exercise is that the community assesses themselves, rather than the facilitator assessing them. Thus this is not about an ‘expert’ coming from outside to assess the community and advise it of its weaknesses and its strengths.

In the next phase, the community chooses three priority practices, relevant to their shared dream, where it feels that it can make progress within a stated timeframe (say, 2–3 months). The discussion evolves around what actions need to be taken in order to reach a next desired level from the current level agreed by the community during self-assessment. For each selected practice, the community itself comes up with certain number of actions, based on its strengths and resources, which would help them reach the target level within the specified period of time. Often specific individuals or groups are identified from the community who take responsibility for each of the actions. To measure the effectiveness of action plan some indicators are defined by the community members.

**Fig. 3:**
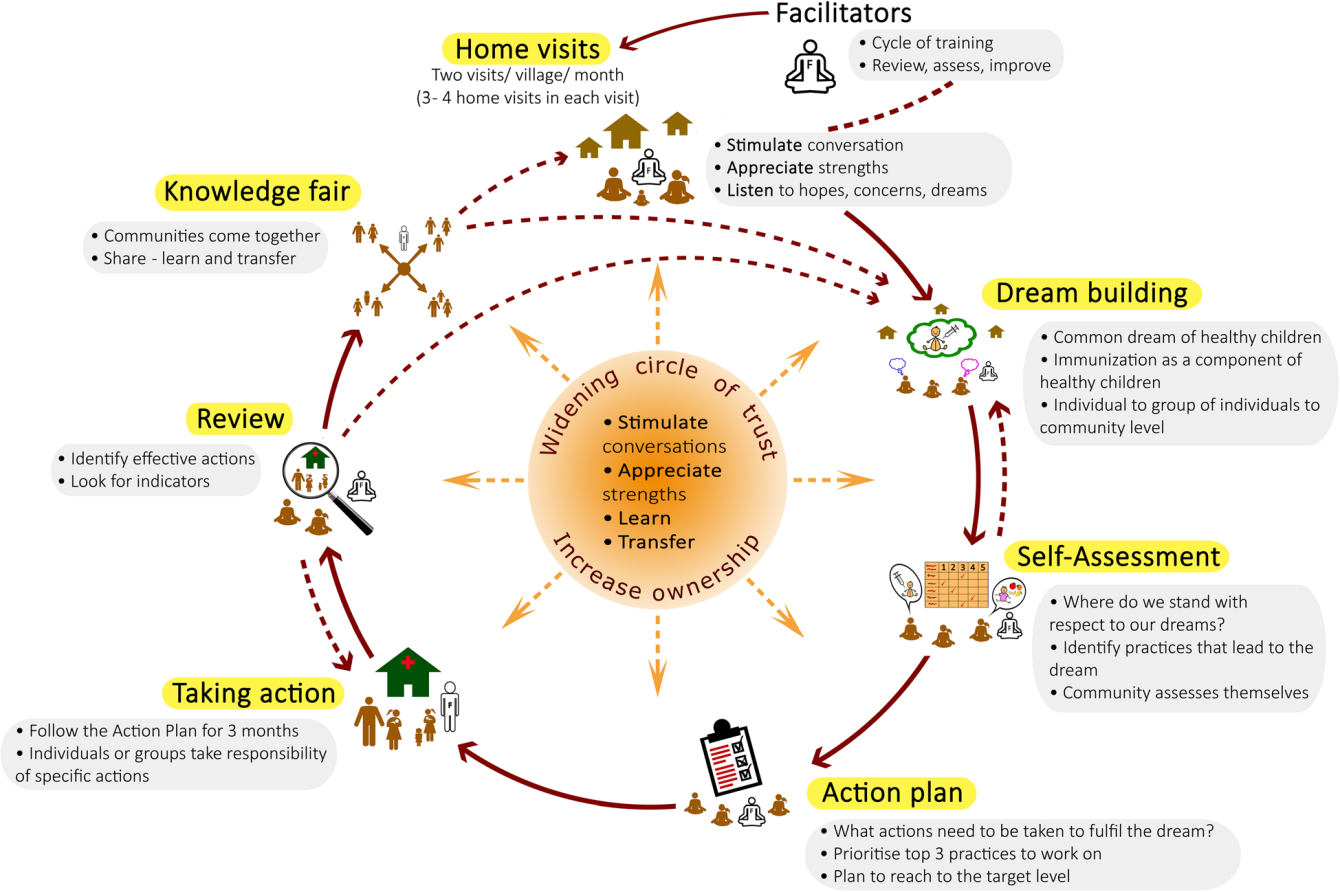
Pictorial illustration of different steps of SALT intervention

This is followed by the action phase and the review process. The emphasis is that a plan is used to help take action, with actions taking precedence. Facilitators then bring communities together to share with and learn from each other in a ‘knowledge fair’ when transfer of knowledge and experience takes place between communities. Depending on the progress and timeline allotted for the intervention, the community might revisit the dream, and the various steps in the process. A pictorial illustration of SALT intervention and its processes are presented in Fig. 3.

### Evaluation outcomes

Our study will assess the intervention’s impact on the following primary outcomes: FIC in children 12–23 months old and coverage rate of three doses of DPT/Pentavalent in 6–23 month old children. As secondary objectives we will study the impact of our intervention on dropout rates between different doses of DPT and OPV, timely immunization, and inequities in immunization coverage by gender, birth order, and various religious and caste groups. In addition, we will explore the intervention’s effect on common childhood illnesses such as diarrhea and acute lower respiratory infection. We will also measure other outcomes which fall on the causal pathway including awareness of immunization service provision in mothers, health service utilization (antenatal check-up and institutional delivery), perception of adherence and completion of immunization schedule in protecting children from diseases, beliefs regarding community engagement, action and practices in the context of child health, and community involvement and ownership.

### Sample size

To calculate the sample size, we considered the two primary outcomes: DPT3 coverage among 6–23 month old children and FIC among 12–23 month age group. Per our initial (pre-baseline) sample size calculation, we needed 120 villages per group, intervention and control, leading to a total of 240 villages, to detect a difference of at least 10 percentage points in coverage between the two groups, with 80% statistical power using a two-sided test at 5% level of significance, after accounting for the correlation in immunization status among children from the same village. We considered an equal allocation of 240 villages across 3 districts, resulting in 40 intervention and 40 control villages in each of the selected districts. Estimates of the coverage rate for DPT3 and FIC in the control group were obtained from the most recent data available at the time (RSOC, 2013–14) [2]. In Assam, the estimates were 65.9% and 55.3% for DPT3 and FIC, respectively. If the coverage rates for DPT3 and FIC in the intervention group are at least 10 percentage points higher, i.e., 75.9% and 65.3%, respectively, our sample size would allow us to detect the difference in coverage rates between the two groups. To calculate the intracluster correlation (ICC) for these outcomes, we extracted DLHS-3 (2007–08) [28] unit level data for Assam and obtained the estimates as 0.21 and 0.25 for DPT3 and FIC, respectively. We further assumed that a village will have a minimum of 15 children 6–23 month old and 10 children 12–23 month old. If there were more than 15 eligible children in a village, we randomly selected 15 children. Baseline survey was conducted in all 240 villages, as per the requirement of pre-baseline sample size calculation.

### Up-to-date ICC and re-estimation of sample size

Sample size is sensitive to the estimate of ICC used in the calculation. ICC, often interpreted as the degree of homogeneity of units within cluster with respect to the outcome variable, is defined as the ratio of between-cluster variability to total variability in the outcome. ICC estimates used in the pre-baseline sample size calculation were based on DLHS-3 (2007–08) data. We expected the recent ICC estimates for DPT3 and FIC to be different, most likely smaller in magnitude because of the improved reach of maternal and child health services, including immunization, under the National Rural Health Mission (NRHM, 2005–2012) [29]. Immunization service delivery, supply chain system, vaccine logistics and the process of linking health system and communities through ASHA- all these have been standardized to a large extent in all rural areas. Moreover, the recent estimates for coverage rate for DPT3 and FIC may have also changed. These changes would affect the sample size needed to detect a difference in coverage rates between the control and intervention groups.

On another note, our intervention is complex in nature, involves sustained interaction over several months with the community. Owing to this intense nature of our intervention, there were concerns that implementation of the intervention may not happen optimally within the timeframe and budget if the sample size was unnecessarily large. The international panel of reviewers of this study also recommended that the sample size and ICC be recalibrated based on new data that would become available to us from the baseline survey. These considerations led us to recalculate the sample size based on recent estimates derived from baseline data.

Table 2 presents the revised estimates based on data from baseline survey and the revised sample size. Note that the ICC estimates based on baseline data have decreased relative to earlier estimates from DLHS-3, as anticipated. On the other hand, vaccination coverage rates have increased substantially relative to RSOC data (2013–14). This sudden increase in vaccination coverage can perhaps be attributed to Mission Indradhanush (MI), a flagship program of the Ministry of Health and Family Welfare (MoHFW) and was also observed in other historically poor-performing states such as Bihar, Rajasthan and Madhya Pradesh [30]. Two phases of MI happened before our baseline survey (phase 1: April–July 2015 and phase 2: Nov 2015-Feb 2016) and all three study districts (Bongaigaon, Kamrup rural, and Udalguri) received at least one round of MI intervention. However, it is possible that this high level of immunization coverage will not be sustained in future after the supplementary immunization activities under MI are discontinued. In view of these high coverage rates, we reduced the minimum detectable difference from 10 to 8 percentage points in the revised calculation of sample size.

**Table 2:** Revised sample size for two primary outcome variables based on SALT baseline survey (July–August 2016) data

Outcome of interest	Updated coverage rate (%)	Updated ICC	Updated sample size (number of villages in each of intervention and control group)
DPT3	84	0.17	57
FIC	79	0.18	90

Per our revised sample size calculation, we needed 90 villages per group to detect a difference of at least 8 percentage points in coverage between the control and intervention groups with 80% statistical power based on a two-sided test having 5% level of significance, after accounting for the correlation in immunization status among children from the same village. Total number of villages required for the study is 180 (90 × 2). We considered an equal allocation of 180 villages across the 3 districts, resulting in 30 intervention and 30 control villages in each of the three selected districts.

### Qualitative component

To evaluate the impact of the intervention, our study includes a qualitative component to complement the quantitative surveys. As a part of qualitative data collection, focus group discussions (FGDs) and key informant interviews (KIIs) were conducted at baseline. The purpose of the FGDs and KIIs was to understand community perceptions regarding immunization, perceptions on immunization of different stakeholders and to gain an understanding of current community engagement if at all.

A more extensive qualitative data collection is planned for endline. FGDs and KIIs will be conducted in a sub-sample of villages in the intervention and control arms that will be representative of villages across the 4 strata identified in three districts. The goal is to examine if, how and why the community mobilized to take ownership of child health and immunization related issues, the perceived benefits and challenges of SALT approach, the perceived improvements in child health with a focus on immunization, and whether the community identifies SALT as a valuable, feasible and sustainable approaches to address community problems.

### Randomization strategy

We adopted a restricted randomization strategy, specifically stratified randomization, to achieve better balance, leveraging information available to us prior to allocation. As opposed to a complete randomized allocation, stratification has the potential to provide explicit balancing of potentially important covariates known to be associated with coverage outcomes, as randomization happens within each stratum [31].

All 240 villages sampled in the baseline survey were randomized to the intervention and control groups. For our stratified randomization strategy, the key advantage of randomization post-baseline survey is that the stratification of villages, and subsequently randomization within stratum, can be based on up-to-date indicators obtained from the baseline data.

Sampled villages from baseline survey, within a district, were stratified into four strata based on a composite score constructed using the following village-level indicators: average number of under-5 children, percentage of HHs living for more than 50 years in the village, percentage of SC HHs, percentage of ST HHs, percentage of Muslim HHs, percentage of HHs belonging to the poorest wealth quintile, percentage of HHs belonging to the richest wealth quintile, percentage of mothers having no formal schooling, percentage of mothers with educational qualification higher secondary or more, percentage of mothers receiving full antenatal care during pregnancy, village had flood last year or not. Full antenatal care is defined as consumption of IFA tablet/syrup for 100+ days, 3+ antenatal check-ups visits, and receipt of at least one TT injection. These indicators were derived based on data from baseline survey.

All indicators were not used for stratification within a district, the choice of variables depended on the district-specific context. For example, in Kamrup rural, all three socio-demographic indicators— percentage of SC HHs, percentage of ST HHs, and percentage of Muslim HHs, were considered whereas for Bongaigaon percentage of ST HHs was not relevant and for Udalguri only percentage of ST HHs was relevant.

To define economic indicators, wealth index was constructed for each household using baseline data. Wealth index is a widely accepted measure of household’s long term economic status [32, 33]. To construct the wealth index, we considered variables related to housing characteristics, sanitation facility of the HH, and asset possession. Each variable is assigned a weight generated through principal component analysis (PCA) and the standardized variables are multiplied by the weights and summed to produce the wealth index. The first principal component, explaining 25% of the total variation in the data, was considered as the wealth index. Based on the wealth index the HHs were divided into quintiles: poorest, poorer, middle, richer, and richest.

Finally, within each stratum we randomized sampled villages to intervention and control groups. After stratification within a district, each stratum contained 20 villages as the baseline sample covered 80 villages in each district. We randomly selected 10 villages to receive the intervention and the remaining 10 villages will continue to receive immunization services from the routine immunization program in place (control group). The flowchart in Fig. 2 illustrates the randomization procedure (in orange). R statistical software was used to implement the randomization strategy through the use of random numbers [34].

### Minimizing intervention-control contamination

In each district, we had randomized the 80 sampled villages to the intervention and control groups in equal proportion. However, according to our revised sample size calculation, we needed only 30 intervention and 30 control villages, that is, we had the liberty to exclude 10 villages from each of the two groups. We attempted to use this opportunity to minimize intervention-control contamination while excluding villages, as opposed to random exclusion.

Transfer of knowledge is a crucial component of SALT intervention. Particularly, during the knowledge fair, the community may have an opportunity to meet peers from the control community and share their knowledge and experience, in case intervention and control villages are located in close proximity. To mitigate potential contamination between intervention and control villages, we attempted to ensure that the intervention and control villages are sufficiently far apart from one another. For our community-based intervention, by separating the intervention and control villages geographically we seek to reduce the chances of interaction between community members belonging to the intervention and control groups. The following procedure was used to ensure geographical buffer between intervention and control villages.

After randomization, we plotted the intervention and control villages on Google Maps by uploading an excel file of villages having locations (defined by “village name, district name, state name”) as a column. Different-color place marks were used for intervention and control villages. Using features of Google Maps, we then calculated straight line distances between intervention and control villages when they visibly appeared to be close by. If the distance is less than 3 km (KM), we discarded one of the villages. While discarding villages, we were mindful about two things: 1) an equal number of intervention and control villages are being discarded and 2) distribution of intervention and control villages across strata does not change significantly as that might have an impact on balance between the two groups.

**Fig. 4:**
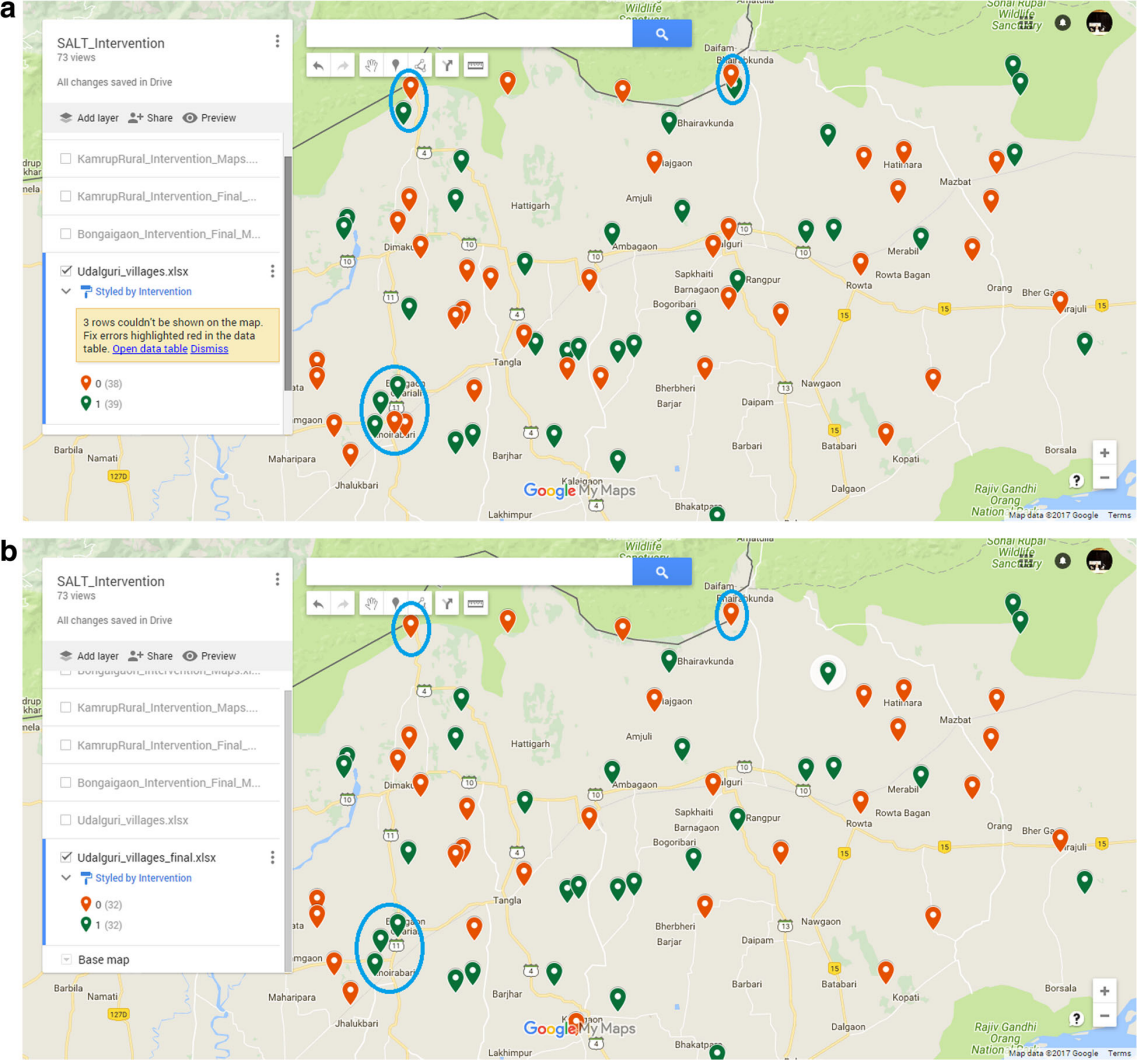
Screen shot of intervention (green) and control (red) villages in Udalguri: Potential scenarios of intervention-control contamination and subsequent use of geographical buffer to minimize it. Source: Google Maps. **a** Original randomization of baseline survey villages. **b** After discarding villages when intervention and control villages are within 3 KM distance

Figure 4 illustrates the procedure using two Google Maps screenshots. In Panel a, we plot all 80 villages from Udalguri district that were randomized to intervention (green) and control (red) groups and in Panel b we present the same plot after discarding villages in instances where intervention and control villages are within 3 KM distance. For example, in bottom left corner, two control villages appeared to be located in close proximity to three intervention villages. Distance calculation confirmed that the straight line distance is less than 3 KM and, hence, two control villages were discarded (circled in blue in Panel a and b). Using the same rationale, two intervention villages were also discarded as seen in the top left and top middle part of the plot (circled in blue in Panel a and b).

### Balancing checks

Balance between intervention and control groups was examined with respect to potentially important covariates after randomization of the 240 villages using baseline data. Although randomization happened at the village level, the unit of analysis is the child. Hence, we performed balancing checks at the level of village, child, child’s household and the mother. Balance was re-examined after excluding the villages discarded to minimize intervention-control contamination. Results in Table 3 suggest that reasonably good balance has been achieved across indicators for villages, children, their HHs, and mothers. This is expected as the randomization of villages to intervention and control groups happened within stratum, after stratifying the villages based on several of the indicators presented in Table 3. The two groups remain balanced after excluding intervention and control villages discarded to minimize intervention-control contamination.

**Table 3:** Balancing checks based on selected village, household (HH), mother and child-level indicators: comparison between intervention and control groups

Characteristics	After randomization of all 240 baseline survey villages			After discarding villages to minimize contamination (180 villages)		
	Control	Intervention	*p*-value*	Control	Intervention	*p*-value*
Village-level indicators						
Village has subcentre	40.5	39.2	0.8334	39.8	41.7	0.7926
Village has secondary school	31.0	25	0.3034	29.6	26	0.5833
Village experienced flood last year	30.2	37.5	0.2359	31.6	35.4	0.5787
% of HHs in village living more than 50 years in same village	75.9	76.5	0.6841	77.2	76.1	0.8800
% of tribal HHs in village	26.4	26.2	0.5359	25.6	27.3	0.6494
% of mothers in village with education HS ^a^ or more	12.3	13.1	0.6777	12.6	13.6	0.6390
% of mothers in village receiving full antenatal care	41.9	42.1	0.936	42.5	43	0.8386
Household-level indicators						
Average HH size	5.33	5.28	0.4336	5.34	5.3	0.4867
Household head is Muslim	27.9	32.3	0.4104	27.7	30.2	0.6453
Household has (pour) flush toilet	35.9	33.6	0.5033	35.3	35.1	0.9965
Household belongs to poorest wealth quintile	19.7	19.7	0.9838	20.4	17.3	0.2405
Average time (in minutes) from household to vaccination site	19.9	20.6	0.5764	19.9	19.9	0.6037
Mother- and child-level indicators						
Mothers received full ANC when pregnant with child	41.9	41.9	0.9612	42.4	42.9	0.8787
Child was born in health facility	85.9	86.5	0.7941	84.9	88.4	0.2184
Child has vaccination card	92.7	92.4	0.7852	92.1	92.1	0.9731
Child (12–23 month old) is fully immunized	78.5	77.6	0.8244	77.6	77.6	0.8479

*Wilcoxon Rank Sum Test with Null Hypothesis: location parameters of the distribution are the same in each group

^a^*HS* higher secondary

To check balances between intervention and control groups based on selected household, mother and child level characteristics we have adjusted for clustering as responses from HHs (as well as mothers and children) within a village are correlated. However, for checking balance based on village level indicators this adjustment does not have any impact on standard errors and hypothesis testing. All analysis has been done using “survey” package [35] in R [36]. Wilcoxon rank sum test has been adjusted as per the survey design [37].

## Discussion

Pre-specification of study protocol for ongoing studies which includes objectives, rationale and methodology of the study increases confidence in the validity of the conclusions and can reduce publication bias [38].

Our study aims to evaluate the impact of a novel community engagement intervention (SALT) in improving childhood immunization uptake. Our community engagement approach is based on the premise that communities can think and act for themselves, and that communities have the capacity to change themselves. It employs facilitated conversations to elicit community strengths, increase self-awareness and stimulate self-confidence and action. Facilitators accompany the community on its path to ownership of the issue of immunization and its solution.

To identify the impact of SALT in increasing immunization coverage, we adopted an experimental design which is considered to be the gold standard for evaluation studies. Specifically, we used a cluster randomized controlled trial design. To recruit clusters and participants at baseline, we used a two-stage stratified random sampling method. Within a district, first we stratified clusters (villages) based on census data and randomly selected villages from each of the four stratum. At the second-stage, we selected random sub-sample of eligible HHs (having children in the age group of 6–23 months) from each selected village. The baseline survey happened during June–August 2016. Post baseline survey, using baseline data we stratified our sampled clusters again and, within each stratum, we randomly allocated them to the intervention and control groups. Randomization after baseline allowed us to use an extensive set of sociodemographic and economic characteristics for stratification at the village level, which were not available from census data. Moreover, it provided us with a more accurate estimate of ICC and coverage rates leading to re-estimation of appropriately justified sample size.

Note that the rationale to consider *stratification* for sampling design is different from the rationale to consider *stratification* for randomization. While the former ensures a representative sample of villages from Assam, having varied socio-demographic characteristics, the latter guarantees balance between intervention and control groups with respect to potentially important covariates at baseline. Since control group forms the basis for counterfactual theory of causation, it is important to ensure that intervention and control groups are similar and our methodology ensures it. A simple comparison of outcomes between sampled children in villages receiving the intervention and those in control villages would allow us to measure change in vaccination coverage outcomes attributable to SALT. The study uses a repeated cross sectional design. Eligible HHs within a cluster were randomly sampled at baseline and HHs will be sampled again at endline following the same method. An appealing feature of repeated cross sectional surveys is that concerns regarding attrition can be avoided [31]. These features of our evaluation design justify the internal validity of our study.

Cluster randomized controlled trail, by design, minimizes the risk of intervention-control contamination as compared to individual-level randomization where sharing of information is more likely to happen if individuals living within the same cluster receive different interventions [39]. However, cluster randomization design does not guarantee that the threat of contamination will be entirely removed. Contamination of control participants may lead to an underestimation of the intervention effect in the context of improving immunization coverage. This may result in rejection of an effective intervention as ineffective because the observed effect size may neither be statistically significant nor programmatically relevant. Our study attempts to minimize intervention-control contamination through introducing distance buffer between intervention and control villages.

Our community engagement intervention; the SALT approach is participatory, interactive, prolonged and involves multiple inter-related steps. RCTs of complex interventions like SALT are often criticised as being ‘black box’ as it can be difficult to know why and how the intervention worked (or not) [40]. At the end of the study, the absence of an impact could be simply because SALT was ineffective in this particular context or it could potentially be because of less than optimal implementation of the intervention. In order to be able to answer this, we will attempt to ‘unpack’ the ‘black box’ by examining underlying processes. To that end, we developed a process evaluation data collection plan for the entire duration of the intervention phase. Specifically, the aim is to answer the following questions: 1) whether the intervention is implemented as intended, 2) whether the intervention incorporates the primary objective of the study (i.e., increasing immunization coverage), 3) consistency of intervention delivery across communities in terms of the process of administering the intervention, 4) how the intervention was received by the HHs and communities across villages and districts, and 5) whether contextual factors influence the implementation of intervention.

As per our revised sample size (90 villages per group), our study is powered to identify a difference of at least eight percentage points in immunization coverage between the intervention and control groups. Any effect size below that may not be detected and, hence, the intervention may be declared as ineffective. It is true that larger the sample size, higher is the chance of detecting a difference, even one that is smaller than the expected difference. We had to tally these methodological considerations against few practical considerations, which also could affect the quality of evidence.

In order to avoid intervention-control contamination, post randomization we discarded some villages from both groups in case intervention and control villages were located in close proximity. It can be argued that this violates the principle of randomization. However, the process of discarding the villages was done in a blinded manner, without considering the logistics of implementation. We assumed that geographical separation of at least 3 KM between intervention and control villages would reduce chances of interaction of community members between groups. Larger distance buffer would have further reduced the chances; however, enforcing a buffer more than 3KM was not feasible since within a district we could discard only 10 villages from each of the two groups.

Lastly, although stratified sampling was used to ensure that selected districts are representative in terms of socio-demographic diversity of Assam, the districts were purposefully selected so that they were not too far away from the central location Guwahati. We should therefore exercise caution while generalizing the study findings to other parts of the state and the country.

## Abbreviations

ACP: AIDS competence process
ANC: Antenatal care
ASHA: Accredited social health activists
BCG: Bacillus Calmette–Guérin
cRCT: Cluster randomized controlled trial
DLHS: District level household and facility survey
DPT3: Three doses of diphtheria, pertussis and tetanus
FGD: Focus group discussion
FIC: Full immunization coverage
GVAP: Global vaccine action plan
HHs: Households
ICC: Intra-cluster correlation
KII: Key informant interview
KM: Kilometers
MI: Mission indradhanush
NRHM: National rural health mission
OPV3: Three doses of oral polio vaccine
PCA: Principal component analysis
RI: Routine immunization
RSOC: Rapid survey on children
SALT: Stimulate, appreciate, learn, transfer
SC: Scheduled caste
SRS: Sample registration system
ST: Scheduled tribe
UIP: Universal immunization program
